# The surgical treatment of familial cylindromatosis through subgaleal scalp excision

**DOI:** 10.3109/23320885.2015.1054392

**Published:** 2015-07-16

**Authors:** Amar Karalija, Magnus N. Andersson

**Affiliations:** 1 ^1^Department of Integrative Medical Biology, Section of Anatomy, Umeå University, SE-901 87 Umeå, Sweden; 2 ^2^Department of Surgical and Perioperative Science, Section of Hand and Plastic Surgery, Umeå University, SE-901 87 Umeå, Sweden

**Keywords:** Cylindromatosis, subgaleal excision, skin cancer, familial cylindromatosis, Brooke-Spiegler syndrome, turban tumor syndrome, head and neck

## Abstract

We treated a 65-year-old woman with familial cylindromatosis, with cylindromas covering the entire scalp. Subgaleal tumor excision and split skin grafting was performed. The graft take was deemed to be excellent, with almost 100% coverage 2.5 weeks after operation, no complications and a satisfying esthetic result.

## Introduction

Familial cylindromatosis (Brooke-Spiegler syndrome; turban tumor syndrome) is a rare autosomal dominant disease, causing a prolific development of tumors originating from the adnexa of the skin [1]. Although benign in most instances, the tumors are more prone to arise in the craniofacial region, causing the patient a great deal of practical and aesthetic discomfort. Furthermore, there is a potential risk of the cylindroma turning malignant and giving rise to cylindromcarcinoma, particularly in the case of multiple scalp cylindromas [2].

Different therapeutic approaches have been attempted including abrasion, radiotherapy and single tumor excision, as well as more extensive scalp excisions with subsequent skin grafting [3]. A concern raised in relation to split skin grafting is the possible fragility of the skin and its ability to support a wig. Due to the well vascularised nature of the scalp, a certain concern over extensive bleeding has also been raised. This report describes the treatment of a 65-year-old female suffering from familial cylindromatosis, and the relatively simple yet successful surgical strategy chosen.

## Case report

**Figure 1.**
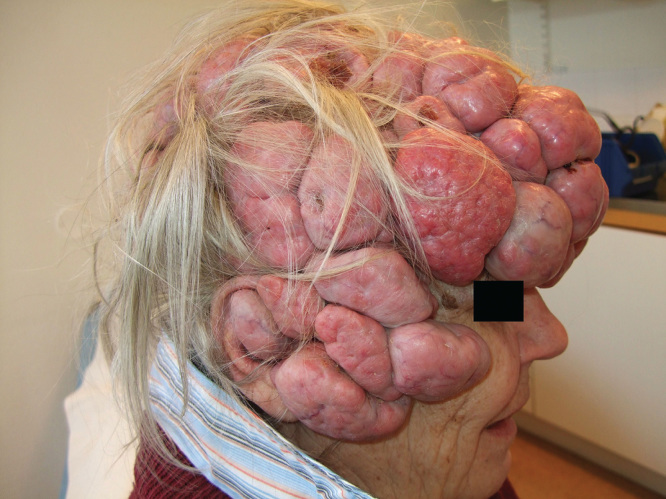
Status before the surgical treatment, showing a scalp virtually completely covered by tumor tissue.

An otherwise healthy, unmedicated woman 65 years of age was referred to the Department of Hand and Plastic Surgery in Umeå University Hospital in 2013. She had suffered from multiple tumors, foremostly present in the scalp and face, for the predominant part of her adult life (Figure 1). Apart from one previous instance of surgical removal of the tumors, the patient was reluctant to seek medical help, instead choosing to avoid social interaction and wearing a hat covering the scalp tumors. According to the patient, several of her close family members suffered from similar tumors. A previous histopathological examination confirmed the diagnosis to be cylindromatosis.

**Figure 2.**
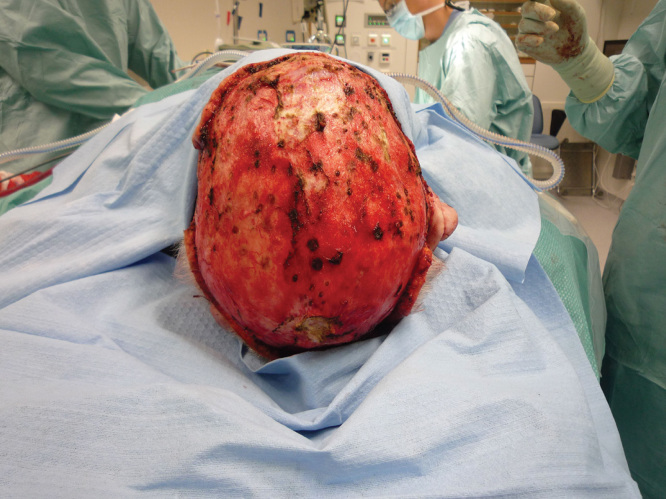
The cranium after subgaleal excision.

We decided to treat the patient with tumor excision and split skin grafting under general anesthesia. In the first session, the skin of the scalp was carefully removed by dissecting to the subgaleal plane and following the avascular compartment (Figure 2). Thorough haemostasis was applied, and a meshed split skin graft was finally used to reconstruct the scalp. The bleeding noted originated from the edges of the excised skin and not from the tumors themselves. In a second session, excision of the skin of the neck and right ear was performed, with subsequent meshed split skin grafting. Additionally, multiple tumors were excised from the torso and legs.

**Figure 3.**
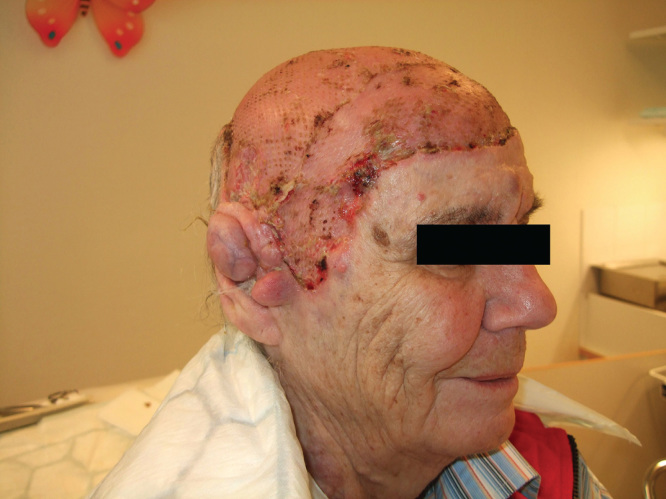
Status 2.5 weeks after the first excision and split skin grafting, showing excellent healing and coverage.

**Figure 4.**
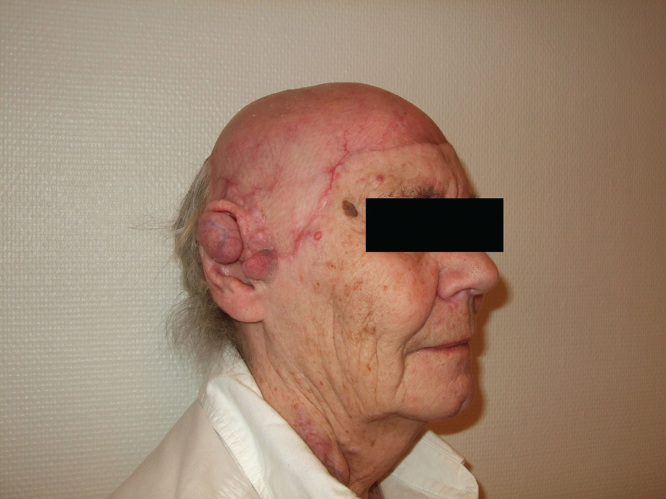
Picture showing the final result, 4 months after the initial procedure.

The patient recuperated well following both operations. Due to anemia, blood was administered after the first procedure, and the patient was quickly mobilized. The graft take after both operations was excellent, with almost 100% coverage after 2.5 weeks (Figure 3). At a later follow-up 4 months after the first procedure, the aesthetic result of the operation was deemed excellent (Figure 4). The patient was eventually supplied with a fitting wig, covering the grafted area. She experienced a great improvement regarding self confidence and social interaction capabilities, with an improved quality of life.

## Discussion

The Brooke-Spiegler syndrome is a very rare autosomal dominant hereditary disease first described in 1892 and 1899 by Brooke and Spiegler, respectively [4,5]. Although benign in most instances, the aesthetically unappealing tumors may cause local pain, hemorrhage, local infections and in some cases even chronic anemia [6]. Furthermore, the cylindroma tumors have a potential of malignant transformation, thereby strengthening the indication for treatment. Therapy with dermabrasion, radiofrequencey devices and YAG laser is unlikely to be sufficient in the treatment of these deep growing tumors [7], making surgery the most appropriate option.

Due to the well-vascularized nature of the cylindroma tumors, surgeons have expressed concern over extensive perioperative and postoperative bleeding. This has prompted some to opt for more advanced approaches such as preoperative radiological embolization of the arteries of the scalp [3]. In treating our subject, we chose a simple strategy. In this case, a subgaleal approach was applied when excising the scalp. This unvascularized structure was followed in order to minimize hemorrhage, and improve the possibilities of radical excision. Indeed, the perioperative bleeding noted originated from the edges of the area of excision, and not from the wound itself. The patient required a single blood transfusion after the first operation and had no need for additional blood after the second procedure. She recuperated well following both operations and was sent home shortly after.

We conclude that subgaleal excision with subsequent split skin grafting is a fairly simple and straight forward approach when treating patients with cylindromatosis. The quality of the skin was sufficient to support a well-fitting wig 4 months after operation, and the overall aesthetical result was satisfactory. Despite certain hemorrhage, the operation was well tolerated by our otherwise healthy subject. In the case described, the therapy greatly contributed to improving the patients self esteem and quality of life.

**Acknowledgements** The authors thank M. Wiberg for his valuable input and support. Authorship statement: Both authors contributed to the creation of this work.

***Declaration of interest:*** The authors report no conflicts of interest. The authors alone are responsible for the content and writing of the paper.
